# Simulation of time-resolved x-ray absorption spectroscopy of ultrafast dynamics in particle-hole-excited 4‐(2-thienyl)-2,1,3-benzothiadiazole

**DOI:** 10.1063/4.0000016

**Published:** 2020-07-06

**Authors:** Khadijeh Khalili, Ludger Inhester, Caroline Arnold, Anders S. Gertsen, Jens Wenzel Andreasen, Robin Santra

**Affiliations:** 1 Department of Energy Conversion and Storage, Technical University of Denmark, Fysikvej 310, 2800 Kgs. Lyngby, Denmark; 2 Center for Free-Electron Laser Science, DESY, Notkestrasse 85, 22607 Hamburg, Germany; 3 The Hamburg Centre for Ultrafast Imaging, Luruper Chaussee 149, 22761 Hamburg, Germany; 4 Department of Physics, Universität Hamburg, Jungiusstrasse 9, 20355 Hamburg, Germany

## Abstract

To date, alternating co-polymers based on electron-rich and electron-poor units are the most attractive materials to control functionality of organic semiconductor layers in which ultrafast excited-state processes play a key role. We present a computational study of the photoinduced excited-state dynamics of the 4-(2-thienyl)-2,1,3-benzothiadiazole (BT-1T) molecule, which is a common building block in the backbone of *π*-conjugated polymers used for organic electronics. In contrast to homo-polymer materials, such as oligothiophene, BT-1T has two non-identical units, namely, thiophene and benzothiadiazole, making it attractive for intramolecular charge transfer studies. To gain a thorough understanding of the coupling of excited-state dynamics with nuclear motion, we consider a scenario based on femtosecond time-resolved x-ray absorption spectroscopy using an x-ray free-electron laser in combination with a synchronized ultraviolet femtosecond laser. Using Tully's fewest switches surface hopping approach in combination with excited-state calculations at the level of configuration interaction singles, we calculate the gas-phase x-ray absorption spectrum at the carbon and nitrogen *K* edges as a function of time after excitation to the lowest electronically excited state. The results of our time-resolved calculations exhibit the charge transfer driven by non-Born-Oppenheimer physics from the benzothiadiazole to thiophene units during relaxation to the ground state. Furthermore, our *ab initio* molecular dynamics simulations indicate that the excited-state relaxation processes involve bond elongation in the benzothiadiazole unit as well as thiophene ring puckering at a time scale of 100 fs. We show that these dynamical trends can be identified from the time-dependent x-ray absorption spectrum.

## I. INTRODUCTION

The sub-femtosecond x-ray pulses provided by x-ray free-electron lasers enable investigations of ultrafast, non-equilibrium dynamics of functional materials as well as their electronic and geometric structure. By exploiting the atomic element specificity from inner-shell transitions as well as the sensitivity of the core-level energies to internuclear separation, x-ray absorption spectroscopy (XAS) is a promising tool to probe local geometric information of molecules.^1–4^ Detailed exploration of ultrafast, nonadiabatic dynamics in photoexcited molecules requires an exploration of the underlying electronic structure changes that drive the structural dynamics. This is feasible by using time-resolved (pump-probe) techniques in which a non-equilibrium state is initiated by a pump pulse and probed by means of a suitable probe pulse arriving with a given time delay.^5–8^

In the last decade, organic photovoltaics (OPVs) have attracted increasing attention as an alternative to silicon-based solar cells due to their low cost and easy manufacturing.^9,10^ However, when upscaled to large-area cells with industrial relevance, OPVs have been suffering from low efficiencies compared to traditional, silicon-based photovoltaics.^11^ Remarkable efforts have been made to increase OPV efficiencies, specifically in terms of materials discovery and optimization; this includes improved alignment of energy levels through substitution, improved charge transfer dynamics through morphology optimization, and improved charge extraction through optimization of interfacial layers, giving rise to a drastic increase in power conversion efficiency, which now exceeds 17%.^12–15^ An early, but key, strategy to increase efficiency has been the lowering of the bandgap of OPV absorber materials for better sunlight harvesting, leading to the synthesis of a wide range of low-bandgap donor co-polymers exhibiting charge-transfer absorption. The backbones of these consist of electron-rich aromatic units, often thiophene derivatives, accompanied by electron-deficient units. This building-block materials design principle has proved advantageous for the screening of new donor materials with improved electronic properties.^16–18^

The functioning of OPV active layer materials is based on the processes in their photoexcited states; therefore, a fundamental understanding of these complex processes and their decay mechanisms can give rise to improvements in the efficiency of such devices. A popular way to study the excited-state relaxation of co-polymers is to study the dynamics of their donor–acceptor (D–A) backbones as oligomeric model compounds. Moreover, D–A *π*-conjugated molecules have attracted attention in the context of optoelectronics, where excited-state deactivation plays a key role. Benzothiadiazole (BT) and thiophene (T), as fundamental units of a family of efficient low-bandgap electron-donating polymers^19–21^ and a variety of polymers with applications in electronic devices,^22^ have already been investigated in a few studies separately or in a *π*-conjugated framework. Iagatti *et al.*^23^ studied the relationship between the structural properties and the excited-state dynamics of dTBT (D–A–D framework based on an electron-withdrawing/accepting BT core connected to two flanking electron-donating T rings) in solution and solid state using *ab initio* calculations and transient-absorption and time-resolved fluorescence measurements. They concluded that the main relaxation mechanism involves an intermolecular charge transfer accompanied by planarization of the molecule's geometry. The same dTBT molecule has been considered as a model compound to study the charge-transfer relaxation in D–A type conjugated materials by Scarongella *et al.*^24^ Using time-resolved femtosecond transient-absorption spectroscopy in solution and in the solid state as well as density functional theory (DFT) calculations, they reported a planar backbone in both the ground and excited states and weak conformational relaxations including marginal contraction of the central benzene ring and elongation of the molecule along the short axis. They have also reported some spectral dynamics in the molecule occurring with time constants of a few picoseconds (ps), which are so far not fully elucidated. The discrepancy in the two reported structural relaxations of the molecule is controversial and requires more investigations. In order to overcome the limitations of optical transient-absorption spectroscopy for revealing the underlying dynamics, we consider time-resolved XAS to obtain complementary information on the excited-state dynamics in D–A type molecules.

**FIG. 1.**
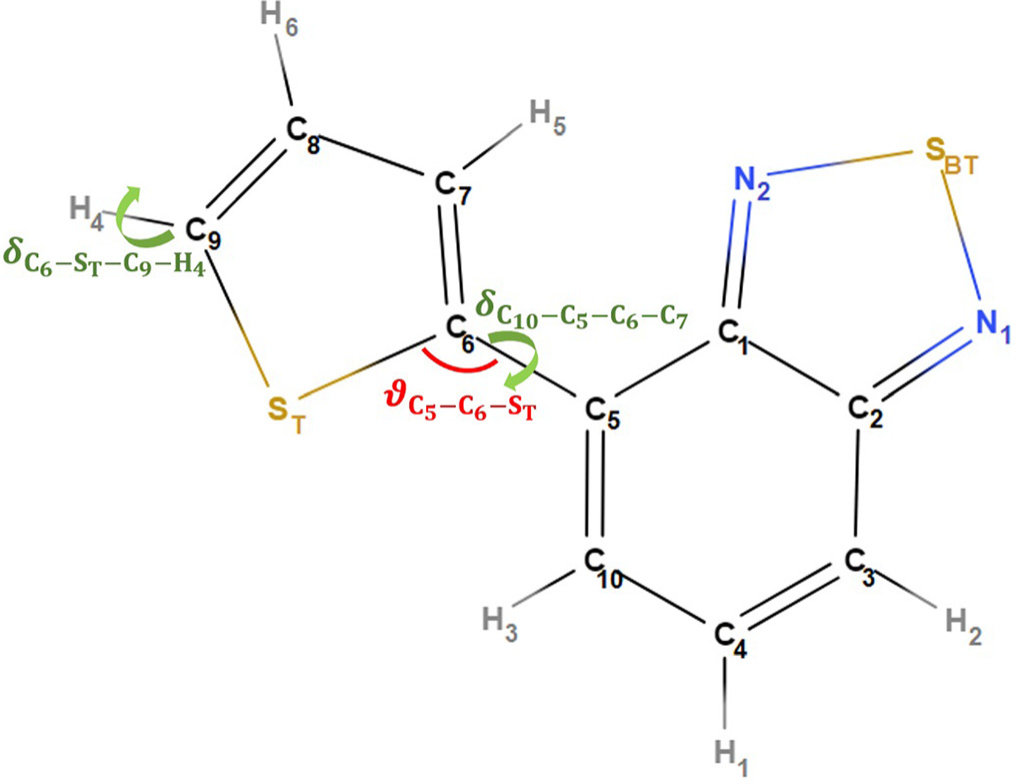
Numbering scheme of BT-1T as addressed in the text (the angles considered in the analysis are also highlighted).

In this paper, we study the excited-state dynamics of 4-(2-thienyl)-2,1,3-benzothiadiazole (BT-1T) molecule, characterized by a D–A structure in which one T ring is connected to one BT unit (Fig. 1). In our previous work,^25^ we showed that there are two conformers, of which the one shown in Fig. 1 is the most energetically favorable. We then investigated the ultrafast charge transfer dynamics following the UV photoionization of this molecule. We showed that the charge transfer can be inspected by time-resolved XAS exploiting its atomic site specificity. Moreover, by ionizing from the highest occupied molecular orbital (HOMO), our earlier findings demonstrated that time-resolved XAS allows us to resolve local geometrical changes in the molecule. Here, we extend our earlier study by considering particle-hole photoexcitation instead of photoionization (pure hole formation), which is more relevant for PV applications. This way, we also strengthen our understanding of the main relaxation processes in the lowest excited state of the BT-1T molecule.

Several time-resolved experiments have been conducted to explore the complex excited-state relaxation dynamics of *π*-conjugated structures.^26–28^ Experimental data are usually combined with theoretical investigations as an indispensable tool for a more detailed description of the observed dynamics. However, it has always been a challenge to theoretically describe excited-state dynamics of a molecule. Nonadiabatic excited-state molecular-dynamics calculations have been performed using time-dependent density functional theory (TDDFT),^29^ algebraic diagrammatic construction to second order,^30,31^ or configuration interaction singles (CIS)^32–34^ to study excited-state deactivation mechanisms via internal conversion or relaxation to the ground state (GS). The choice of the level of theory strongly depends on the kind of molecule, its size, and desired properties to investigate. Moreover, the results are sensitive to other factors. For instance, the popular TDDFT is known to be functional dependent and can lead to inaccurate excitation energies and state inversions;^35^ the affordable CIS is size-consistent and free of self-interaction errors, but lacks dynamic and non-dynamic correlation effects beyond the correlation between particle and hole. Still, if a suitable basis set is chosen, CIS can provide a reasonably accurate excited-state description.^36^ Therefore, the choice of the excited-state computational method is crucial because the failures in, for instance, incorrect state ordering, poor distribution of oscillator strengths, and erroneous descriptions of the excited-state potential energy surfaces can give rise to misleading computed absorption spectra.^31,35^ In the present work, we systematically investigate the photoabsorption spectra of the relevant D–A molecule, BT-1T, in the gas phase by using Tully's fewest switches surface hopping (FSSH) approach^37–39^ based on excited-state calculations at the level of CIS. We carefully verify that the employed CIS method provides a computationally efficient and qualitatively accurate description of the XAS in the energy region we are interested in. In our work, we do not consider relaxation mechanisms due to intersystem crossing, which are expected to play a role on time scales from tens to hundreds of picoseconds^40–43^ and, thus, potentially could also play a relevant role for the ultrafast relaxation of BT-1T.

## II. COMPUTATIONAL DETAILS

### A. Nonadiabatic molecular dynamics

Throughout this work, the FSSH approach is employed to describe the nonadiabatic, ultrafast dynamics of BT-1T after photoexcitation. FSSH is based on a quantum-mechanical treatment of the electronic subsystem, while the nuclear subsystem is treated classically, propagating along a classical trajectory. In these classical trajectories, the nuclei move on a single excited-state adiabatic potential energy surface. Nonadiabatic effects are incorporated by allowing the nuclei to hop between the surfaces, based on the nonadiabatic hopping probability. An ensemble of 100 nuclear initial conditions (i.e., coordinates and momenta) is obtained by quasiclassical sampling^44,45^ of the normal-mode coordinates of BT-1T in the ground state. Each of the 100 initial conditions is then independently propagated with a time step of 0.5 fs and a total propagation time of 400 fs.

### B. Electronic-structure calculations

Electronic-structure data, energies, energy gradients, and nonadiabatic couplings are determined on the fly at the level of CIS using the XMOLECULE toolkit.^46^ In the CIS method, the electronic wavefunction is approximated by a linear expansion of electron configurations,

$$|\Psi \rangle =\sum_{a,r}C_{a}^{r}|\Phi_{a}^{r}\rangle ,$$ (1)

where $\left|\Phi_{a}^{r}\right\rangle$ are the determinants with single particle-hole excitations relative to the Hartree–Fock (HF) ground state with amplitudes $C_{a}^{r}$. The particle-hole determinant describes an excitation from *occupied* molecular orbitals (MOs) labeled by *a*,*b*, ⋯ to *virtual* orbitals labeled by *r*,*s*, ⋯. As a consequence of the *Brillouin theorem*, which states that there are no coupling matrix elements between the ground state and the single excitation (or $\langle \Phi_{0}|\hat{H}|\Phi_{a}^{r}\rangle =0$), the CIS matrix has a block structure with the HF ground state as an eigenstate of $\hat{H}$.^47^ The ground-state block in the CIS matrix gives the restricted Hartree–Fock (RHF) ground-state energy, $E_{0}=\langle \Phi_{0}|H|\Phi_{0}\rangle$. The single-excitation block elements in the MO space are

$$\left\langle \Phi_{b}^{s}|H|\Phi_{a}^{r}\rangle =(\varepsilon_{r}-\varepsilon_{a})\delta_{a,b}\delta_{r,s}-(rs|ba)+2(ra|bs\right),$$ (2)

with (rs|ba) and (ra|bs) being two-electron integrals. The resulting matrix is then diagonalized to find the eigenvalues as the electronic excitation energies in the CIS method. The corresponding CIS energy gradient is given by

$$\begin{array}{c} \frac{dE_{\text{CIS}}}{\partial R}=\sum_{a,r}\sum_{b,s}{C_{b}^{s}}^{*}C_{a}^{r}(\frac{d\varepsilon_{r}}{dR}-\frac{d\varepsilon_{a}}{dR})\delta_{a,b}\delta_{r,s}\\ +\sum_{a,r}\sum_{b,s}{C_{b}^{s}}^{*}C_{a}^{r}(-\frac{d(rs|ba)}{dR}+2\frac{d(ra|bs)}{dR}). \end{array}$$ (3)

In our approach, the energy gradients are computed analytically based on the coupled-perturbed Hartree–Fock theory^48–50^ as implemented in XMOLECULE.^25^

### C. Photoabsorption cross sections

Our method for evaluating x-ray absorption cross sections was described in detail elsewhere.^25,51^ The absorption line strengths are essentially determined by the transition dipole moments between the involved CI eigenvectors. Within the CIS expansion, these are given for a transition between the ground state and an excited state as $\sum_{a,r}C_{a}^{r}\langle \Phi_{0}|\hat{\mu }|\Phi_{a}^{r}\rangle$ and for a transition between excited states as $\sum_{a,r}\sum_{b,s}{C_{a}^{r}}^{*}C_{b}^{s}\langle \Phi_{a}^{r}|\hat{\mu }|\Phi_{b}^{s}\rangle$ (μ=x,y,z). We discuss the accuracy of the calculated absorption spectra for valence and core and core +valence excitation in the electronic supplementary material. Calculated oxygen *K*-edge x-ray absorption spectra of formaldehyde in Fig. S3 in the electronic supplementary material show that by considering an absolute shift in the spectrum, the CIS method (with a relatively small basis set) provides a qualitatively correct spectrum in the energy range covering the first few absorption lines (higher absorption lines are out of the scope of the current study).

## III. RESULTS AND DISCUSSION

The lowest excited-state transition energies, oscillator strengths, and the values of the dominant CIS coefficients for BT-1T in gas phase are shown in Table I. Table I compares the results from the CIS calculation using XMOLECULE to two other levels of theory, namely, complete-active-space self-consistent field (CASSCF) and complete-active-space second-order perturbation theory (CASPT2), using Molcas 8.2^52^ (restricted-active-space state interaction (RASSI)^53^ is used to compute oscillator strengths). All calculations are performed using the 6–31G basis set, which is justified by a basis-set analysis as a reasonable compromise between computational cost and precision within the CIS level of theory for describing the lowest-energy transition (cf. Sec. I in electronic supplementary material). We emphasize that the present work should be seen as an initial effort to highlight the possibilities of x-ray absorption spectroscopy for investigating ultrafast chemical dynamics and charge transfer processes.

**TABLE I.** Vertical transition energies for the $S_{1}$ state calculated from three different levels of theory. The transition energy (*E*), oscillator strength (*f*), and the HOMO–LUMO expansion coefficients (*C*) are reported.

	E(eV)	*f*	C
CIS	4.08	3.88$\;\times 10^{-1}$	0.92
CASSCF/RASSI	4.61	4.07 $\times 10^{-1}$	0.81
CASPT2/RASSI	3.93	4.07 $\times 10^{-1}$	0.81

**FIG. 2.**
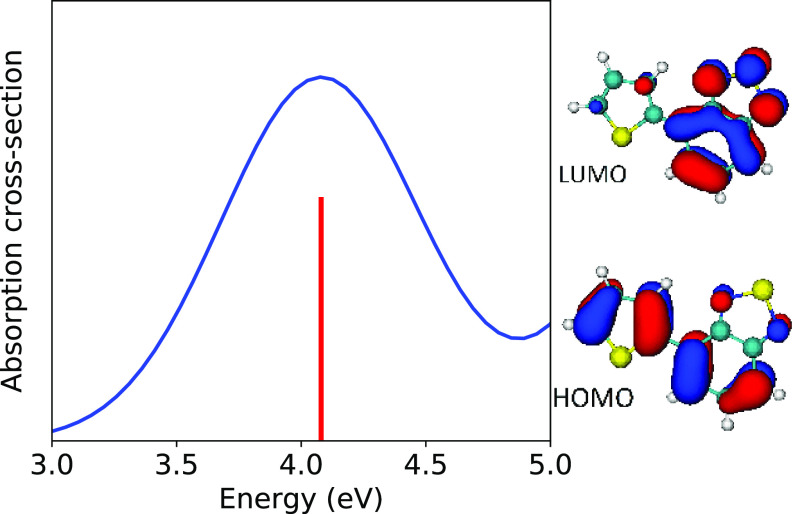
Absorption of BT-1T in gas-phase calculated at CIS/6-31G level (vertical red line for the energy position of absorption at the ground-state optimized geometry). The images on the right show the HOMO and LUMO molecular orbitals that are involved in the first excitation, $S_{0}\to S_{1}$. (The orbitals are visualized using VMD-1.9.^54^)

In Table I, the first absorption band in the gas-phase electronic spectrum of BT-1T is attributed to a low-lying absorbing singlet state ($S_{1}$) dominated in all three calculations by the HOMO→LUMO particle-hole configuration. The UV-absorption spectrum of BT-1T is shown in Fig. 2 together with the HOMO *π* and LUMO $\pi^{*}$ orbital. While the LUMO is mostly localized on the BT unit with some contribution on the BT sulfur, $S_{\text{BT}}$, the HOMO is distributed over both parts (BT and T) and has specifically very low contributions at the two sulfur atoms.

### A. Electronic state population

**FIG. 3.**
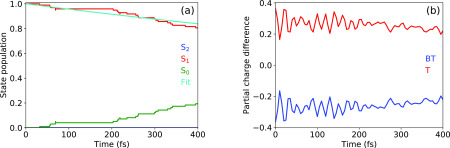
Time evolution of (a) the population of the ground and first two excited states as well as the fit curve for $S_{1}$ population and (b) the partial charge difference for trajectories started in the $S_{1}$ state (difference between ground- and excited-state charges).

In order to investigate the nonadiabatic excited-state dynamics of BT-1T, trajectories were initiated from the first absorption band populating the $S_{1}$ state. The evolution of the state populations for trajectories initiated in $S_{1}$ is shown in Fig. 3(a). As one can see, the $S_{1}$ state decays relatively slowly such that only 20% of the trajectories relax to the ground state within 400 fs. The simulations do not show any upward hops, leading to population of the $S_{2}$ state. By fitting an exponential decay to the population of the $S_{1}$ state, we obtain a lifetime of the first excited state of 2.27 ps. Figure 3(b) shows the dynamics of the charge population in the BT-1T molecule after initial excitation to the $S_{1}$ state from the time-dependent Mulliken charge population.^55^ The plot shows the partial charge difference between ground and excited states as a function of time. As expected from Fig. 2, excitation to the $S_{1}$ involves a charge transfer that creates an excess negative charge on the BT unit and an excess positive charge on the T unit. During the relaxation to the $S_{0}$, the excess charges reduce and both curves tend to zero value (ground state difference is almost zero).

### B. Structural dynamics

As mentioned in the Introduction, there has been a discrepancy in results reported in literature regarding the structural relaxation after photoexcitation of benzothiadiazoles coupled to thiophenes. In 2013, Scarongella *et al.*^24^ studied the CDTBT and dTBT molecules using a combination of transient absorption (TA) spectroscopy and density functional theory (DFT) calculations. The former of these consists of a dTBT unit flanked by a phenyl group on one side and a carbazole on the other. For this CDTBT, both TA measurements and M06-2X/6-31+G(d) optimizations of the ground- and first excited states in vacuum suggested that the molecule undergoes a planarization around the BT-thiophene bonds. They report, however, based on M06-2X/6-31+G(d) or M06-2X/6-31G(d,p) (ambiguous), that the dTBT molecule is flat, i.e., has 0° torsions in both the ground- and first excited states which is surprising given the large similarities between the CDTBT and dTBT molecules. We have, hence, repeated the calculations using the same levels of theory, also in Gaussian 09,^56^ and found ground state torsional angles of ∼16° for dTBT, very close to the 15° that they themselves report for CDTBT. Our optimized structures were all confirmed to be minima by Hessian analyses.

Three years later, in 2016, Iagatti *et al.*^23^ reported transient absorption and time-resolved fluorescence measurements in a range of solvents showing that di-thiophene substituted benzothiadiazole (dTBT) undergoes a planarization between the BT and the thiophenes on a time scale of ∼1 ps upon excitation. These observations were substantiated by B3LYP/def2-TZVP calculations using COSMO to model solvent effects, which yielded ground state torsional angles between BT and the thiophenes of 7–14° from planarity depending on the conformer, while the optimized S_1_ states were found to be completely planar. They conclude that the large Stokes shift observed experimentally for dTBT can be ascribed to exactly this planarization.

In the present work, we do not consider solvent effects, and our geometry for BT-1T is based on HF/6-31G calculations in vacuum. Although adding polarization functions to the basis, i.e., HF/6-31G(d), induces a torsion in the BT-1T ground state of 26° (both geometries were confirmed to be minima at their chosen level of theories by Hessian analyses), the more than one order of magnitude increase in computational time upon going from 6-31G to 6-31G(d) is prohibitive for the dynamical calculations presented herein. We are aware that capturing the structural planarization, which is shown experimentally to happen on time scales of around one picosecond in solvents,^23^ could very well be critical also in the gas-phase, but we emphasize that the key focus of this work is the development of a framework to follow the ultrafast molecular charge transfer dynamics following excitation and that the implementation of methods for more accurate structural descriptions is ongoing. The discussed limitations in the modeling of the structural dynamics of the molecule do not restrict us in gaining knowledge about the interplay of structural dynamics and time-dependent changes in the XAS.

**FIG. 4.**
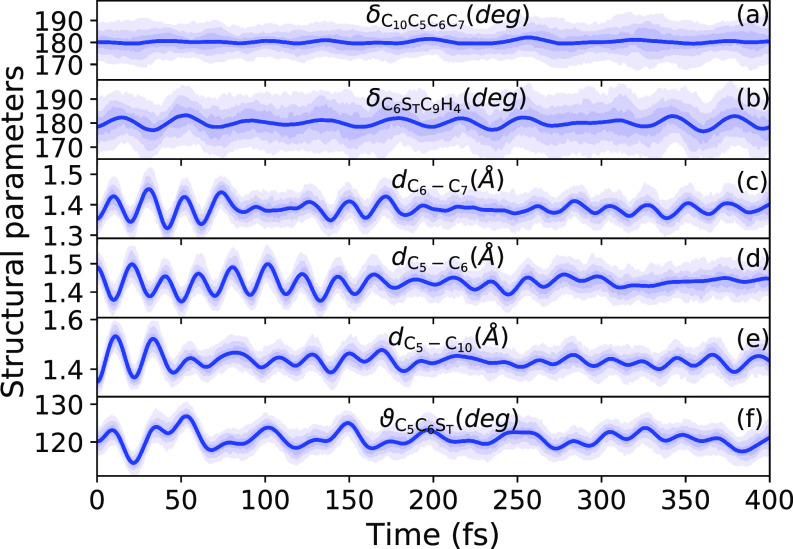
Time evolution of selected bond lengths and bond angles after excitation to the $S_{1}$ state. The shaded areas indicate the regions between percentiles of the distribution (12.5%–87.5%, 25%–75%, and 37.5%–67.5%). The solid line shows the mean value.

Figure 4 shows the dynamics of selected geometrical features as a function of time. Specifically, the torsional angle $\delta_{C_{10}{-C}_{5}{-C}_{6}{-C}_{7}}$ (shown in Fig. 1 as a criterion for the rotation of the thiophene unit) and the related bonds (for possible double bond shift or ring puckering), $C_{6}{-C}_{7},\;C_{5}{-C}_{6}$ and $C_{5}{-C}_{10}$, are chosen. Moreover, we show the structural parameters $\vartheta_{C_{5}{-C}_{6}{-S}_{T}}$ and $\delta_{C_{6}{-S}_{T}{-C}_{9}{-H}_{4}}$ (see Fig. 1) to discuss the possible out-of-plane motions in the molecule. As can be seen in Figs. 4(a) and 4(b), the initial excitation induces small fluctuations in the dihedral angle $\delta_{C_{6}{-S}_{T}{-C}_{9}{-H}_{4}}$, while the torsional angle $\delta_{C_{10}{-C}_{5}{-C}_{6}{-C}_{7}}$ stays rather constant. This constant torsion is, as discussed above, most probably an artifact of the chosen basis set, and planarization effects could indeed be relevant for longer time scale simulations with basis sets including polarization functions if these were feasible. The C−C bond lengths in Figs. 4(c)–4(e) undergo oscillations with slowly decaying amplitudes, where the bonds $C_{6}{-C}_{7}$ and $C_{5}{-C}_{10}$ oscillate anticyclic to $C_{5}{-C}_{6}$ at very short time delays, which indicates a flip between single and double bonds between the corresponding units. At larger time delays, we observe an elongation of $C_{5}{-C}_{10}$ and a shortening of the two other bonds. The $\vartheta_{C_{5}{-C}_{6}{-S}_{T}}$ angle [shown in Fig. 4(f)] oscillates around the ground-state value with an amplitude of 6°. Based on the results from Fig. 4, we conclude that excited-state relaxation leads to a ring puckering in the thiophene unit.

**FIG. 5.**
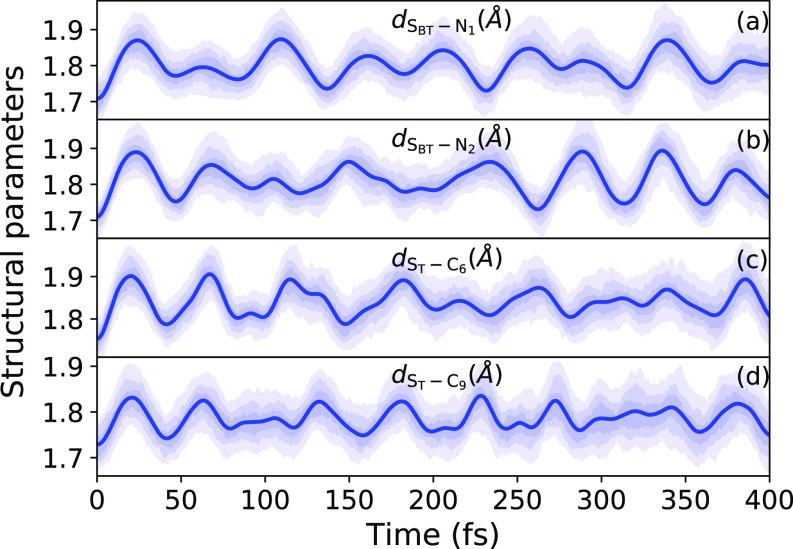
Time evolution of $S_{\text{BT}}-N$ and $S_{T}-C$ bond lengths after excitation to the $S_{1}$ state. The shaded areas indicate the regions between percentiles of the distribution (12.5%–87.5%, 25%–75%, and 37.5%–67.5%). The solid line shows the mean value.

We further inspect the time evolution of structural features relevant to the chemical environment of the two sulfur atoms that are the $S_{\text{BT}}–N$ and $S_{T}–C$ bond lengths. Figures 5(a)–5(d) show the excitation induced vibrations of the respective bonds. Moreover, for $S_{\text{BT}}–N$ and $S_{T}–C$, we see a slight-bond length increase after excitation.

### C. Time-resolved x-ray absorption spectrum

**FIG. 6.**
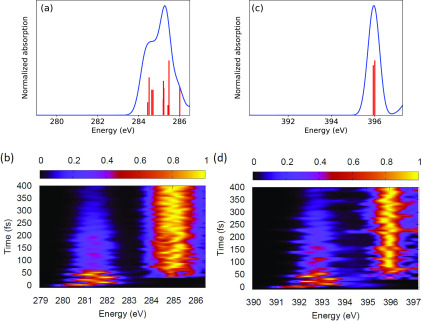
Normalized carbon and nitrogen *K* edge XAS intensity vs photon energy for (a) and (c) the ground state (red lines represent oscillator strength for vertical transitions at ground-state optimized geometry) and for (b) and (d) different time delays after photoexcitation to the $S_{1}$ state. The calculated C *K* edge spectra are shifted by −11 and −16 eV based on the analysis reported in the electronic supplementary material.

We now turn to the time-resolved (pump-probe) x-ray absorption spectroscopy and discuss its ability to obtain detailed information on the previously discussed dynamical evolution underlying the excited-state relaxation dynamics. Figures 6(a)–6(d) provide the carbon and nitrogen *K* edge spectra, respectively, revealing instantaneous changes upon photoexcitation. The figures show the spectra in the energy range around the first absorption resonances of the excited state (ES) XAS at the respective edge. In Figs. 6(a) and 6(c), each transition line without broadening is shown with a red vertical line. From left to right, the lines can be attributed to excitation of the core levels in $C_{10},\;C_{4},\;C_{3},\;C_{7},\;C_{9},\;C_{8},\;C_{5},\;C_{2},\;C_{1}$, and $C_{6}$ for the C *K* edge in Fig. 6(a) and $N_{1}$ and $N_{2}$ for the N *K* edge in Fig. 6(c). Two main dynamical features can be seen in the time evolution of the absorption spectra in Figs. 6(b) and 6(d). In both figures, an excited-state absorption (ESA) peak appears immediately at energies ∼5 eV below the first GS absorption band. This ES low-energy contribution energy position oscillates with an amplitude of ∼1 eV for ca. 100 fs. At larger time delays, the ESA peaks show subtle variation in amplitude with smaller changes in the position of the peaks and eventually decreases due to the depletion of excited state population. At the same time, the corresponding increase in the GS features is observed. Due to the clear separation of ground-state and excite-state absorption features, the decay of excited-state absorption feature can be directly associated with the non-Born–Oppenheimer transition to the ground state (see Fig. 3), which goes hand in hand with a transfer of charge. The oscillations of the spectral shifts can be associated with changes of the partial charges in the vicinity of the absorbing atoms,^57,58^ which are linked to oscillations in the bond lengths.

**FIG. 7.**
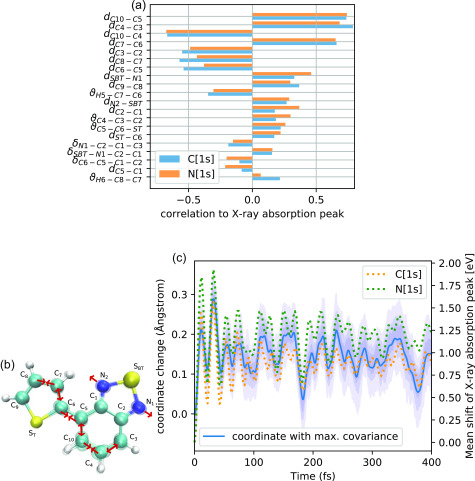
(a) Correlation coefficients between internal coordinates and shifts in the excited-state x-ray absorption peak below the C- and N-edge (270–284 eV and 385–395 eV, respectively). The 20 most correlated internal coordinates are shown. (b) Coordinate that maximizes the covariance between geometry and spectral peak shifts. (c) Time evolution of the coordinate shown in subfigure (b) compared with the evolution of the mean peak position of the excited-state x-ray absorption features. The shaded areas indicate the regions between percentiles of the distribution (12.5%–87.5%, 25%–75%, and 37.5%–67.5%).

The relation of the structural dynamics of the molecule to oscillations of x-ray absorption peaks is further elucidated in Fig. 7. Combining all the sampled geometries and calculated x-ray absorption lines from all trajectories up to a time of 400 fs, we have conducted a correlation analysis employing a complete set of internal coordinates. In Fig. 7(a), we report the correlation coefficients of the 20 internal coordinates with largest correlation to the two absorption peak shifts. As can be seen, the strongest (anti-)correlation to both absorption peaks is consistently observed for carbon-carbon bond stretches. In particular, strong correlation is indicated for the $C_{10}{-C}_{5},\;C_{4}{-C}_{3},\;C_{7}{-C}_{6}$ bond length and anti-correlation for the $C_{10}{-C}_{4},\;C_{3}{-C}_{2},\;C_{8}{-C}_{7},\;C_{6}{-C}_{5}$ bond length. To further explain the relation of the dynamics in the high-dimensional coordinate space to the shifts in the x-ray absorption, we have computed the collective coordinate that has the largest covariance with the two discussed absorption peak shifts via a partial-least-square regression analysis.^59^ This way, we have identified a collective motion that can explain almost 70% of the variation in the two x-ray absorption peak positions while constituting only a minor part (ratio of variations 2%) of the overall geometrical variations in the simulation data. The resulting coordinate is illustrated in Fig. 7(b). As depicted in the drawing, the coordinate mainly involves a concerted elongation of the $C_{4}{-C}_{3},\;C_{10}{-C}_{5},\;C_{6}{-C}_{7}$ bond and contraction of the $C_{5}{-C}_{6},\;C_{10}{-C}_{4},\;C_{8}{-C}_{7}$ bond, which is accompanied by a bending of the $N_{1}{-S}_{\text{BT}}{-N}_{2}$ angle. In Fig. 7(c), the time evolution projected onto this coordinate is compared with the evolution of spectral shifts. This comparison clearly demonstrates that within the first 100 fs there is a strong correlation of shifts in the x-ray absorption peaks with the described vibrational motion in the molecule. For longer times, one can see that the distribution in the coordinate indicated by the shaded area becomes broader, resulting in the depletion of the absorption peak oscillations. The (anti)-correlated pattern of vibrations shown in Figs. 7(a) and 7(b) can be understood from the shape of the HOMO orbital (see Fig. 2). Having a significant *π* character along the $C_{3}{-C}_{4},\;C_{5}{-C}_{10},\;C_{6}{-C}_{7},\;C_{8}{-C}_{9}$ bonds, the HOMO orbital energy is sensitive to stretches in these bonds. Because the x-ray absorption transitions that we are considering here predominantly populate the electron hole in the HOMO orbital of the excited state, the described vibration results in a shift in the excited state x-ray absorption resonance. We can, therefore, conclude that the observed oscillations in the x-ray absorption peaks at the C and N edges are to a large degree a result of the vibrational dynamics along the collective coordinate in Fig. 7(b) due to its impact on the valence-hole orbital energy.

Similar to the discussed results for C and N *K*-edge absorption, we have also conducted calculations on the time-dependent x-ray absorption spectrum around the sulfur *K* and $L_{2,3}$ edges (cf. Fig. S11 in electronic supplementary material). Because the HOMO orbital has a very low population around the two sulfur atoms the transition dipole between the HOMO orbital and the sulfur core orbitals is very low. At the same time, there is only a small energetic separation between ground state absorption features and excited state features that can be attributed to a refilling of the HOMO orbital. These facts impede a clear interpretation of changes in excited-state absorption spectra in terms of excited-state dynamics. We, therefore, conclude that information about the structural changes is best retrieved from the C and N *K*-edge spectrum.

## IV. CONCLUSION

With respect to the necessity of a deeper understanding of photophysical properties of conjugated oligomers/polymers for a successful design of new photovoltaic materials and architectures, we have systematically investigated the excited-state dynamics of the 4–(2-thienyl)-2,1,3-benzothiadiazole (BT-1T) molecule. We have verified that the employed level of electronic-structure modeling, CIS, is able to qualitatively reproduce low-lying absorption-band features reasonably well compared to experiments. For the energy window around and a few eV below the low-lying absorption bands, we have performed time-resolved x-ray absorption spectroscopy simulations to investigate excited-state ($S_{1}\to S_{0}$) relaxation processes occurring on an ultrafast time scale for BT-1T in gas phase. We find that the nonadiabatic transition from the excited to the ground state is associated with electron transfer from the benzothiadiazole unit to the thiophene unit. We demonstrate that this charge transfer can be followed via transient excited state absorption resonances. Moreover, structural changes leave a fingerprint in the C *K*-edge and N *K*-edge XAS spectra. Our work shows how a specific excited-state bond elongation shifts the binding energy of the initially created valence hole and, thus, results in oscillations in the x-ray absorption spectrum.

Knowledge of the excited-state relaxation pathways and time scales in photoexcited organic materials is essential for detailed insights into the charge-carrier localization and the dynamics, which have a strong effect on the efficiency of optoelectronic devices based on such materials. Such information clarifies whether charge-transfer relaxation competes with the functional processes on the same time scales in an organic solar cell. Here, we have offered a theoretical methodology addressing time-resolved x-ray absorption experiments to gain the essential information, which can be used, for instance, for designing a new synthetic route or new conjugated materials for high efficiency OPVs. The implementation of more precise quantum chemical methods for describing charge-transfer excitations into XMOLECULE is ongoing.

## SUPPLEMENTARY MATERIAL

See the electronic supplementary material for a systematic analysis supporting the CIS XAS spectrum results, as mentioned in the text.

## Data Availability

The data that support the findings of this study are available from the corresponding author upon reasonable request.
